# Employment, Volunteering, and Health‐Related Resource Use in Anti‐Amyloid in Asymptomatic Alzheimer's Disease (A4) Study

**DOI:** 10.1002/alz70860_105779

**Published:** 2025-12-23

**Authors:** Carolyn W. Zhu, Charlene Flournoy, Rema Raman, Mary Sano

**Affiliations:** ^1^ James J. Peters VA Medical Center, Bronx, NY, USA; ^2^ Icahn School of Medicine at Mount Sinai, New York, NY, USA; ^3^ Alzheimer's Therapeutic Research Institute, Keck School of Medicine, University of Southern California, San Diego, CA, USA

## Abstract

**Background:**

“Pre‐symptomatic” Alzheimer's disease (AD), defined by the presence of brain amyloid in the absence of cognitive symptoms, has been identified as an ideal stage to target for the prevention of progression to cognitive impairment and dementia, but little is known about the health economic impact (including the impact on the ability to work and health‐related resources) of this disease stage. Here we compare changes in health economic outcomes over time in cognitively unimpaired older adults with evidence of amyloid accumulation (A4 Study) to otherwise matched participants who did not meet subthreshold levels of amyloid accumulation (LEARN).

**Method:**

Health‐related resource use was self‐reported by participant and/or study partner using the Resource Use Inventory (RUI), including participation in paid employment, volunteer work, any use of hospitalization, doctor visit, and paid or unpaid help. All study visits where the RUI was assessed were included. Multivariable logistic regression models for panel data were used to estimate the relationship between main independent variables of study arm (A4 vs. LEARN), time (months since baseline), and their interaction and RUI items. Other variables included screening MMSE, age, sex, race/ethnicity, and education.

**Result:**

At baseline, A4 participants (*N* = 1165) were older (71.9±4.8 vs. 70.5±4.3), had lower MMSE (28.8±1.3 vs. 29.0±1.2) and more likely to have at least 1 APOEε4 allele (59.0% vs. 22.5%) than LEARN (*N* = 507). There were no differences in years of schooling (16.6±2.8), sex (∼60% female), race/ethnicity (∼90% white). All RUI items were similar except for lower rate of volunteering in A4 (57.9%) when compared to LEARN (65.5%).

Multivariable analyses results showed that controlling for covariates, A4 participants were at baseline more likely than LEARN to participate in paid employment (OR±SE=1.886±0.598) but less likely to volunteer (OR±SE=0.448 ±0.117). Likelihood of participation in paid employment decreased each month (OR±SE=0.964±0.011). There were no between‐group differences in the rate of change in participation in any outcomes over time.

**Conclusion:**

The RUI was able to capture subtle decreases in the rate of employment over time. Results suggest little detectable impact of amyloid levels on rate of change over time in paid employment, volunteering, and health‐related resource use at preclinical stage.

